# Activation of Cytosolic Calpain, Not Caspase, Is Underlying Mechanism for Hypoxic RGC Damage in Human Retinal Explants

**DOI:** 10.1167/iovs.61.13.13

**Published:** 2020-11-06

**Authors:** Momoko Kobayashi-Otsugu, Kana Orihara, Emi Nakajima, Thomas R. Shearer, Mitsuyoshi Azuma

**Affiliations:** 1 Senju Laboratory of Ocular Sciences, Senju Pharmaceutical Corporation Limited, Portland, Oregon, United States; 2 Department of Integrative Biomedical & Diagnostic Sciences, Oregon Health & Science University, Portland, Oregon, United States

**Keywords:** calpain, caspase-3, retinal explant, hypoxia/reoxygenation, human, monkey

## Abstract

**Purpose:**

Activation of proteolytic enzymes, calpains and caspases, have been observed in many models of retinal disease. We previously demonstrated calpain activation in monkey retinal explants cultured under hypoxia. However, cellular responses are often species-specific. The purpose of the present study was to determine whether calpains or caspase-3 was involved in retinal ganglion cell (RGC) damage caused by hypoxia/reoxygenation in human retinal explants. The explant model was improved by use of an oxygen-controlled chamber.

**Methods:**

Human and monkey retinal explants were cultured under hypoxic conditions in an oxygen-controlled chamber and then reoxygenated. Calpain inhibitor SNJ-1945 was maintained throughout the culture period. Immunohistochemistry and immunoblotting were performed for calpains 1 and 2, calpastatin, α-spectrin, calpain-specific α-spectrin breakdown product at 150 kDa (SBDP150), caspase-3, and apoptosis-inducing factor (AIF). Propidium iodide (PI) staining measured membrane disruption, and TUNEL staining detected DNA fragmentation.

**Results:**

Activation of calpains in nerve fibers and increases of PI-positive RGCs were observed in retinal explants incubated for 16-hour hypoxia/8-hour reoxygenation. Except for autolysis of calpain 2, SNJ-1945 ameliorated these changes. In longer incubations under 24-hour hypoxia/16-hour reoxygenation, TUNEL-positive cells appeared, although activated caspase-3 and truncated AIF were not observed. DNA fragmentation was inhibited by SNJ-1945.

**Conclusions:**

An improved human retinal explant model showed that calpains, not caspase-3, were involved in cell damage induced by hypoxia/reoxygenation. This finding could be relevant for patient treatment with a calpain inhibitor if calpain activation is documented in human retinal ischemic diseases.

Retinal ganglion cells (RGCs) in the inner retina transmit photoreceptor information to the brain via their long axonal extensions that comprise the optic nerve.^1^^,^^2^ Ischemia is known to injure the optic nerve,^3^ and optic nerve degeneration is associated with RGC death.^4^^,^^5^ RGC death is common in central retinal artery occlusion, glaucoma, and diabetic retinopathy and leads to visual impairment and blindness.^6^^–^^9^

In neuronal cells deprived of oxygen and glucose, dysregulation of ion channels and transporters leads to accumulation of cytosolic calcium.^10^ Excess calcium ion opens the mitochondrial permeability transition pore (mPTP)^11^ and releases cytochrome c into the cytosol.^12^^,^^13^ The apoptotic protease activating factor 1 (Apaf-1) then oligomerizes to form the large apoptosome complex. Apoptosome recruits and activates pro-caspase-9, initiating activation of executioner caspase-3 (EC 3.4.22.56) and leads to apoptosis.^14^^,^^15^

Calpains (EC 3.4.22.52/53) are an another superfamily involved in apoptosis, and they are calcium-dependent proteases found in retinal tissues.^16^^,^^17^ The ubiquitously expressed conventional calpains 1 and 2 have cysteine catalytic subunits, are inhibited by an endogenous specific inhibitor calpastatin,^18^ and conduct limited proteolysis of specific substrates such as α-spectrin.^19^^,^^20^ Mitochondrial calpain 1 also cleaves the apoptosis inducing factor (AIF) to truncated form (tAIF), which can translocate to nucleus.^21^^,^^22^ Thus proteolysis by calpains in the cytoplasm and mitochondria are involved in a caspase-*in*dependent cell death pathway.

Cell death pathways are complex and often cell- and species-specific. Furthermore, activation of calpains and caspases have been implicated in many in vivo and in vitro models of retinal cell death.^16^^,^^23^^–^^27^ For example, monkey retinal explants showed hypoxia-induced calpain activation in the nerve fiber layer (NFL). This was followed by retinal ganglion cell (RGC) death and was inhibited by synthetic calpain inhibitor SNJ-1945.^26^ However, calpain activation in human retina cannot be extrapolated from such studies in monkeys because of the significant amounts of calpastatin in human retina.^28^ Thus the purpose of the present study was to determine whether calpains or caspase-3 were involved in RGC damage caused by hypoxia in retina from human eyes.

## Materials and Methods

### Human and Monkey Eyes

Twenty-two human eye globes from 20 donors, between eight and 95 years of age, were obtained from the Lions Vision Gift (Portland, OR, USA) using informed consent and are exempt from IRB review. The wide age range was unavoidable for ethical reasons, but acceptable for the purposes of our experiments (see below). Enucleated eyes were cooled and used for experiments between 11 and 27 hr after the death. All experimental procedures complied with the tenets of the Declaration of Helsinki for Ethical Principles for Medical Research Involving Human Subjects.

Comparison globes were also obtained from rhesus macaques (*Macaca mulatta*) at one to nine years of age from the Oregon National Primate Research Center (Beaverton, OR, USA). The wide range of age was unavoidable because they became available from experiments that were unrelated to the present studies. These eyes were acceptable for the purpose of our experiments. The eyes were dissected within two hours after death, and excised eyes were soaked in cold Hanks’ balanced salt solution (HBSS; Corning, Corning, NY, USA). Experimental animals were handled in accordance with the Research in Vision and Ophthalmology Statement for the Use of Animals in Ophthalmic and Vision Research and with the Guiding Principles in the Care and Use of Animals (Department of Health Education and Welfare Publication National Institutes of Health 80-23).

### Retinal Explant Culture

Retinal explant culture was performed as in our previous report,^26^ except for induction of hypoxia. The previous gas-generating pouch system exhibited poor pH control after 24 hours. In the present experiments, atmosphere in the hypoxic chamber (Coy Laboratory Products, Grass Lake, MI, USA) was controlled with a 95% N_2_/5% CO_2_ gas mixture, which maintained a neutral pH in the culture medium. Retinas were dissected into fan-shaped explants with six or seven petals (>5 mm) in HBSS. Each explant was placed with the RGC side facing up on a Millicell organotypic standing insert (0.4 µm, 30 mm diameter; Merck, Darmstadt, Germany) in six-well, culture plates. They were cultured for three hours at 37°C under 95% N_2_/5% CO_2_ in Neurobasal-A medium supplemented with B27, N2 supplements, and 2 mM L-glutamine (Thermo Fisher Scientific, Waltham, MA, USA), and 100 µg/mL primocine (Invivogen, San Diego, CA, USA). Retinal explants were then chamber-cultured under hypoxic conditions for 16 or 24 hours in culture medium with 0.5 mM glucose, under 0.3% oxygen. The retinas were then reoxygenated for eight or 16 hours in culture medium with 5.5 mM glucose. Normoxic control retinas were cultured for 24 or 40 hours in culture medium with 5.5 mM glucose. When present, calpain inhibitor SNJ-1945 in 0.1% dimethyl sulfoxide (DMSO; VWR Life Science, Radnor, PA, USA) was used at a final concentration of 100 µM in the medium.

### Protein Extraction From Retinal Explants and Immunoblotting

Each retinal explant was sonicated in buffer containing 20 mM Tris (pH 7.5), 5 mM EGTA, 5 mM EDTA, 2 mM dithioeryrthritol (DTE), and protease inhibitors (Complete Mini-EDTA-free; Roche Diagnostics, Basel, Switzerland). Protein concentrations in lysates were measured by bicinchoninic acid (BCA) assay (Pierce BCA Protein Assay Kit, Thermo Fisher Scientific) using bovine serum albumin as the standard. For immunoblotting, 5 or 8 µg of each sample was loaded and run on 4% to 12% gradient or 10% SDS-PAGE gels (NuPAGE Bis-Tris Protein Gels; Thermo Fisher Scientific) with 2-(N-morpholino)ethanesulfonic acid buffer or 3-(N-morpholino)propanesulfonic acid buffer. Proteins were then electrotransferred to polyvinylidene fluoride membrane at 100 V for 1 or 1.5 hours. Membranes were then blocked with 5% skim milk in Tris-buffer saline solution (Bio-Rad Laboratories, Hercules, CA, USA) containing 0.05% Tween 20. Each membrane was probed with primary antibodies against α-spectrin (1:2000 dilution; Enzo Life Sciences, Farmingdale, NY, USA), calpain-specific α-spectrin breakdown product at 150 kDa (SBDP150) (1:1000 dilution),^19^ calpain 1 (1:1000 dilution; Thermo Fisher Scientific), calpain 2 (1:500; GeneTex, Irvine, CA, USA), calpastatin (1:500 dilution; Santa Cruz Biotechnology, Dallas, TX, USA), caspase-3 (1:1000 dilution; Cell Signaling Technology, Danvers, MA, USA), AIF (1:500 dilution; Abcam, Cambridge, UK) and β-actin (1:1000 dilution; Merck). For calpain 1 and α-spectrin, immunoreactivity was visualized with secondary antibody conjugated to alkaline phosphatase and the AP Conjugate Substrate Kit (Bio-Rad Laboratories). For other proteins, immunoreactivities were visualized with secondary antibodies conjugated with rabbit (Santa Cruz Biotechnology) or mouse horseradish peroxidase enzyme (GE Healthcare, Chicago, IL, USA) and ECL Plus detection reagents (GE Healthcare). Images of membranes were captured with FluorChem FC2 imager (Cell Biosciences, Santa Clara, CA, USA). Band intensities for α-spectrin, calpain 1, and calpastatin were measured with ImageJ 1.51s (NIH, Bethesda, MD, USA). To compensate for staining variability between membranes, densities of bands were normalized to the density of a β-actin loading control. To determine calpain activation, the calpain 1 autolytic band at 76 kDa was measured. The two-step autolysis produces 78 and 76 kDa bands and reduces Ca^2+^ concentrations required for activation, and these forms have been observed on SDS-PAGE.^29^ In contrast, autolyzed calpain 2 bands at 79 and 78 kDa cannot be resolved from the intact 80 kDa polypeptide by SDS-PAGE.^30^

### Immunohistochemistry of Retinal Sections

Formalin-fixed, paraffin-embedded, 5 µm sections of retinal explants were subjected to immunohistochemistry. The sections were incubated in antigen retrieval buffer (10 mM citrate buffer, pH 6.0; Diagnostic Biosystems, Pleasanton, CA, USA) for 10 minutes at 90°C, after being deparaffinized and rehydrated. For permeabilization, sections were incubated with PBS containing 0.2% Triton-X 100 for 15 minutes. After blocking with PBS containing 10% goat serum (Abcam) for 30 minutes, the sections were incubated with primary antibodies diluted 1:100 against α-spectrin, calpain 1 and 2, and calpastatin (same antibodies used for immunoblotting described above) overnight at 4°C. The sections were washed with PBS three times and were incubated with secondary antibodies conjugated with Alexa Fluor 488 or 568 (1:200 dilution; Thermo Fisher Scientific) together with Hoechst 33342 dye (1:500 dilution; Thermo Fisher Scientific) for visualizing the nuclei. After the sections were washed with PBS three times, mounting agent (Prolong Gold Antifade reagent; Thermo Fisher Scientific) was added and sealed with a coverslip. Images were taken with a fluorescence microscope with the LD A-Plan 20 × 0.3 NA objective lens (Axio Observer 7; Carl Zeiss, Oberkochen, Germany).

### Flat Mount Staining of Retinal Explants

Formalin-fixed retinal explants were incubated overnight at −30°C in DMSO-ethanol (1:4 dilution) to promote penetration of antibodies. Retinal explants were subjected to two cycles of freezing and thawing between −80°C and room temperature in 100% ethanol for 20 minutes. Then retinas were rehydrated in 70%, 50%, and 15% ethanol and PBS for 20 minutes each and incubated overnight with 0.2% Triton X-100. Samples were stained with antibodies for SBDP150 as described above and β-III tubulin (as a neuron marker, 1:500 dilution; Merck). The NFL was observed at approximately 5 mm from the tips of the fan-shaped retinal flat mounts with a model SP5 AOBS spectral confocal system (Leica, Wentzler, Germany) or a model LSM 780 spectral confocal system (Carl Zeiss, Oberkochen, Germany).

### TUNEL, PI Staining, and RGC Counting

To detect cleaved DNA in RGCs, flat mounts of formalin-fixed retinal explants were subjected to the terminal deoxynucleotidyl transferase dUTP nick end labeling assay (TUNEL; In Situ Cell Death Detection Kit, fluorescein; Roche Diagnostics). RNA-binding protein with multiple splicing (RBPMS) as RGC marker^31^ was costained using specific antibody (1:100 dilution; PhosphoSolutions, Aurora, CO, USA) for quantitative measurement. RBPMS and TUNEL-positive cells in ganglion cell layer were counted in four images from two square areas (775 × 775 µm) each at 3 to 5 mm and at 5 to 7 mm away from the tips of the fan-shaped retinal flat mounts. Measurements were averaged, and the percentage of TUNEL-positive RGCs was calculated as [the number of TUNEL and RBPMS-double positive cells/the number of all RBPMS-positive cells] × 100.

To measure disruption of the membrane barrier function, the membrane integrity dye propidium iodide (PI) was used.^32^ After hypoxia/reoxygenation treatment, retinal explants were incubated with a final concentration of 2 µL PI/mL medium for 20 minutes. Flat mount preparations, RBPMS staining, and cell counting were performed as described above. The percentage of PI-positive RGCs were calculated as [the number of PI and RBPMS-double positive cells/the number of all RBPMS-positive cells] × 100.

### Incubation of Normal Retinal Proteins With Calcium, With/Out Human Calpain 1

To investigate proteolysis of substrates by endogenous and exogenous calpains in the test tube, noncultured normal human retinas were sonicated in buffer containing 20 mM Tris (pH 7.5), 5 mM EGTA, 5 mM EDTA, 2 mM DTE. The lysate was then incubated with 10 mM CaCl_2_ with or without 249 µg/mL human calpain 1 (erythrocyte, Merck) for 30 minutes or two hours at 37°C. To confirm proteolysis by calpain, a final concentration of 100 µM SNJ-1945 was also added to some lysates. Proteolysis was terminated by addition of EGTA at a final concentration of 10 mM. Immunoblotting was performed as described above.

### Statistical Analysis

At least three independent experiments from different human and monkey eyes were conducted for all studies in this report. Statistical analyses were performed by the Wilcoxon rank sum test (JMP 12.2.0 statistical software; SAS Institute Inc., Cary, NC, USA). *P* < 0.05 was considered statistically significant.

## Results

### The Localization of Calpain to Specific Layers of Human Retina

**Figure 1.**
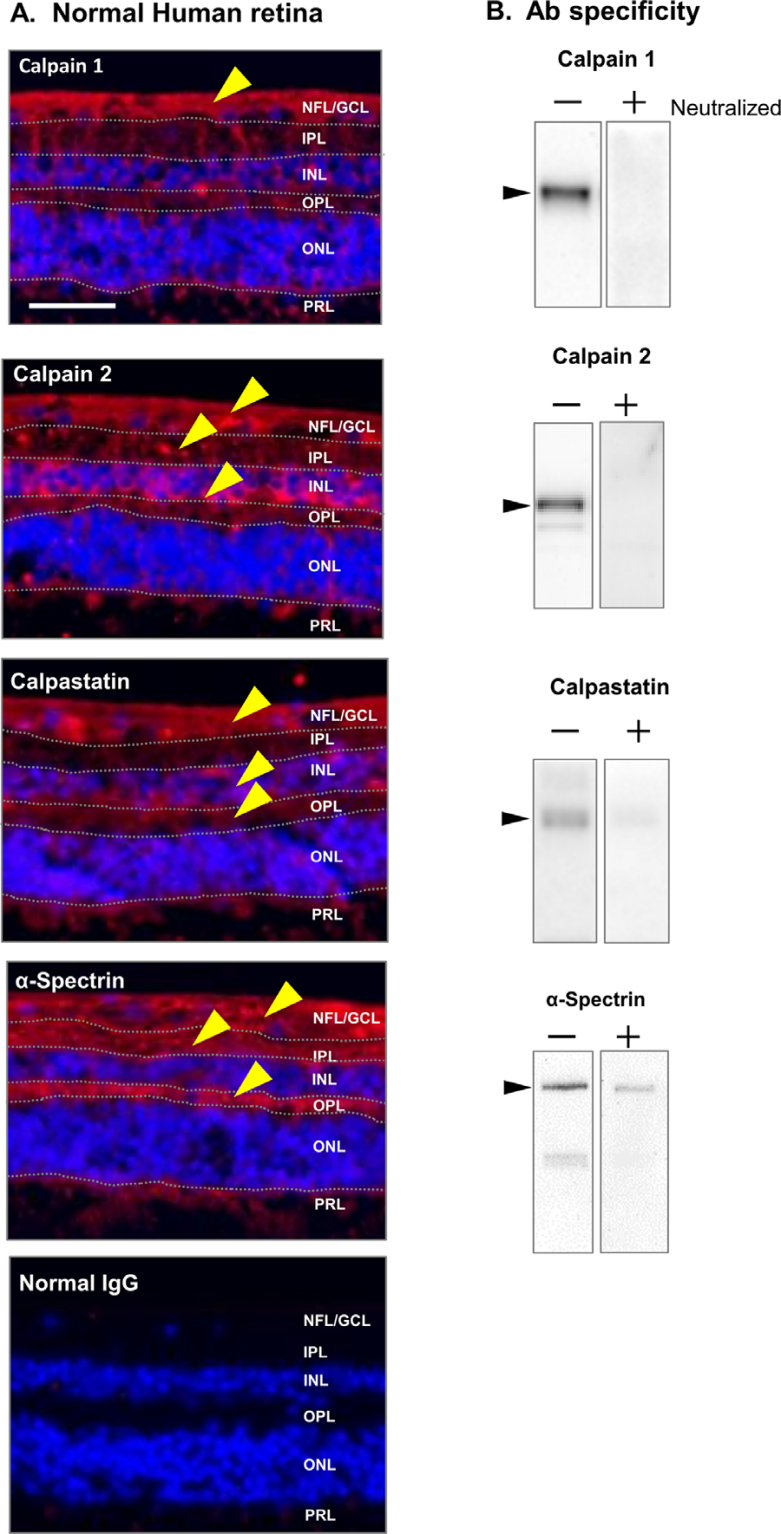
(**A**) Immunohistochemistry of noncultured human retina showing the localization of calpains and related proteins (*red*). All slides were co-stained with Hoechst 33342 nuclear stain (*blue*). Negative staining controls used nonimmunized IgG in place of the specific antibodies. *Scale bar*: 50 µm. NFL, nerve fiber layer; GCL, ganglion cell layer; IPL, inner plexiform layer; OPL, outer plexiform layer; ONL, outer nuclear layer; PRL, photoreceptor layer. *Yellow arrowheads* show the layer expressing each protein. (**B**) Immunoblotting of noncultured human retina showing the specificity of each antibody used for immunohistochemistry in (**A**), before (−) and after (+) neutralization with excess immunizing peptide.

In normal human retina, the protein components of the calpain system were localized in specific layers (Fig. 1A). Low calcium-requiring calpain 1 was localized in the nerve fiber/ganglion cell layers (NFL/GCL, yellow arrowhead). High calcium-requiring calpain 2 was observed in the NFL/GCL, inner plexiform layer, and inner nuclear layer (INL). Endogenous inhibitor calpastatin was located throughout the retina. The α-spectrin substrate was also located throughout the retina but was intense in the NFL/GCL. Negative control immunostaining with normal IgG in place of specific antibodies showed negligible staining (Fig. 1, bottom panel). The localization of the component proteins for the calpain system in these cross-sections of human retina was similar to results obtained from monkey retina.^26^

The antibodies for calpains 1 and 2, calpastatin, and α-spectrin stained as fairly intense, monospecific bands (with minor fragments) when immunoblotted against the total proteins from normal human retinal lysates (“−”, Fig. 1B). Staining from these bands was severely attenuated when the antibodies were first incubated with their immunizing peptide (**“+”**, Fig. 1B). These results indicated that the antibodies were monospecific. In summary, the proteins for a calpain system, complete with two ubiquitous calpains, a natural specific inhibitor, and known substrates were especially prevalent in the neurons in the NFL/GCL in normal human retina. The studies below tested whether these calpain proteins became proteolytically active during hypoxia in human retinal explants.

### Calpain, Not Caspase, Is Activated in Hypoxic Retina

**Figure 2.**
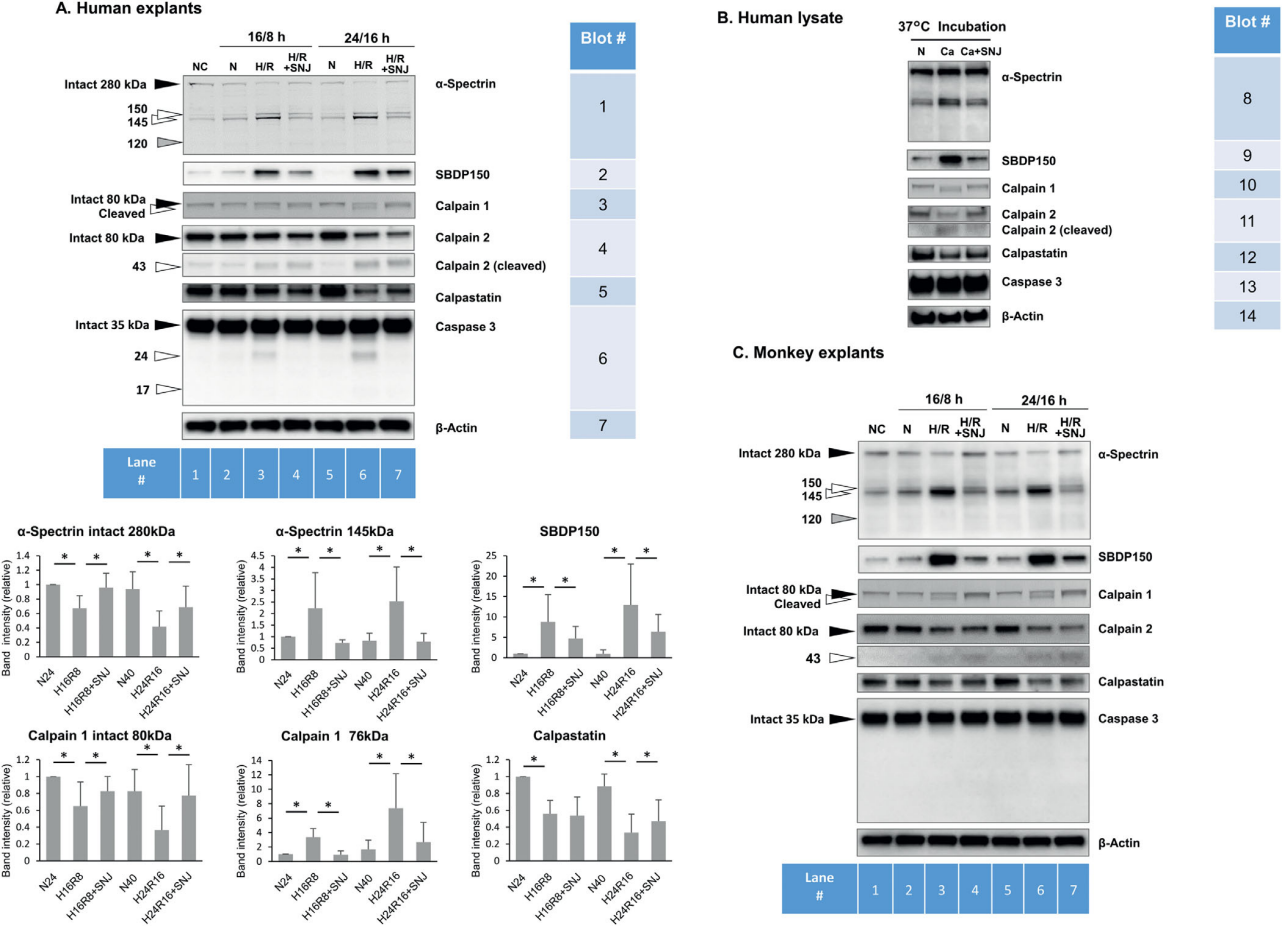
(**A**) Activation of calpain, but not caspase-3, in human retinal explants under hypoxia/reoxygenation. *Black* and *white arrowheads* show bands for intact and cleaved proteins, respectively. *Gray arrowheads* show the expected breakdown product by caspase-3. Endogenous β-actin was used as the loading control. The *bar graphs* at the bottom show the band intensities relative to N24 (normoxia for 24 hours) for α-spectrin, calpain 1, calpastatin and the proteolyzed fragments. Data are means ± SD (n = 5). *P < 0.05 (Wilcoxon rank sum test). (**B**) Immunoblot for lysates from noncultured human retina incubated in the test tube for 2 hours without CaCl_2_ (N), with CaCl_2_ (Ca), or with CaCl_2_ plus 100 µM SNJ-1945 (Ca + SNJ). (**C**) Monkey retinal explants under hypoxia/reoxygenation. 16/8 hours, hypoxia for 16 hours followed by reoxygenation for 8 hours; 24/16 hours, hypoxia for 24 hours followed by reoxygenation for 16 hours; NC, noncultured; N, normoxia; H/R, hypoxia and reoxygenation; H/R+SNJ, hypoxia and reoxygenation with 100 µM SNJ-1945.

To test for calpain activity, explants were first subjected to hypoxia followed by reoxygenation. The entire explants were then homogenized and immunoblotted. Using an antibody against endogenous α-spectrin in retina revealed that the intensity of the band for intact α-spectrin migrating at 280 kDa decreased during hypoxia/reoxygenation (Fig. 2A, Blot no. 1, lanes 3 and 6; graph at bottom). The α-spectrin is a well-known calpain substrate,^20^ and the intensity of bands for spectrin breakdown products (SBDP) at 150 and 145 kDa increased with hypoxia/reoxygenation (Blot no. 1, lower portion; graph). As confirmation, another antibody that was specific for the calpain-specific SBDP150 fragment was used as a marker for calpain activation, and this band was likewise increased by hypoxia/reoxygenation (Blot no. 2, lanes 3 and 6; graph). No obvious differences in proteolysis of α-spectrin were observed between young and old human retinas (data not shown).

Ubiquitous calpains show autolytic N-termination truncation leading to activation.29,30 Hypoxia/reoxygenation caused the appearance of autolyzed calpain 1 (Blot no. 3, lanes 3 and 6; graph), indicating calpain 1 activation. Hypoxia/reoxygenation caused loss of intact calpain 2 (80 kDa) and formation of degradation fragment at 43 kDa (Blot no. 4, lanes 3 and 6)—also indicative of calpain 2 activation.^33^ Endogenous calpain inhibitor calpastatin is a suicide substrate of calpains.^34^ The abundant band for calpastatin in human explants was decreased after hypoxia/reoxygenation (Blot no. 5, lanes 3 and 6; graph).

The synthetic calpain inhibitor SNJ-1945 (Fig. 2A, lanes 4 and 7) ameliorated the degradation of endogenous α-spectrin (Blot no. 1 and graph), increase of calpain-specific SBDP150 (Blot no. 2; graph), autolysis of calpain 1 (Blot no. 3; graph), and loss of calpastatin (Blot no. 5; graph).

Hypoxia/reoxygenation did not seem to activate caspase in these human explants caspase-3 (Blot no. 6). Neither the active forms of caspase-3 at 17 and 12 kDa,^35^ nor the caspase-specific 120 kDa α-spectrin breakdown products were observed after hypoxia/reoxygenation (Blot no. 1). A calpain-dependent fragment of caspase-3 at 24 kDa^36^ was slightly increased after hypoxia/reoxygenation (Blot no. 6, lanes 3 and 6), but this fragment was inhibited by calpain inhibitor SNJ-1945 (lanes 4 and 7). Also, except for a slight increase in caspase-3 fragment, a repeat of the above experiments using explants from monkeys (Fig. 2C) produced similar results to those from human retina. In both species, longer hypoxia/reoxygenation resulted in more severe proteolysis (Fig. 2A and 2C, “16/8 h” vs. “24/16 h”).

Overall, the data indicated that calpain, not caspase, is activated in hypoxic retinal explants. The incubation conditions used to test this conclusion seemed valid because no proteins bands changed during the total 40-hour incubation period using normoxic medium (Figs. 2A and 2C, lanes 1 vs. 5). Furthermore, the culture media maintained the original neutral pH-indicating color even after 24-hour culture of human retinal explants in the hypoxic chamber. This was an improvement over the previous method using gas-generating pouch system, which did not regulate pH well.

The findings above were interesting because they indicated that hypoxia/reoxygenation stressors were potent enough to raise calcium levels high enough to activate calcium-dependent calpains and for the calpains to overcome the abundant amounts of endogenous inhibitor present in human explants. To further confirm calpain activation, the homogenized lysates from noncultured human retina were incubated with extremely high levels (10 mM) of calcium (Fig. 2B). Calpain-induced proteolysis of α-spectrin (Blot nos. 8 and 9), autolysis of calpains (Blots nos. 10 and 11), loss of calpastatin (Blot no. 12), and inhibition by SNJ-1945 were observed. These changes were similar to those observed after hypoxia/reoxygenation in retinal explants, except that SNJ-1945 inhibited autolysis of calpain 2 (Fig. 2B). Thus calpain activation in hypoxic explants from human and monkey retinas is likely due to an influx of calcium from the medium into at least some layers of retina.

### Hypoxia/Reoxygenation Activated Calpain in the Nerve Fiber Layer

**Figure 3.**
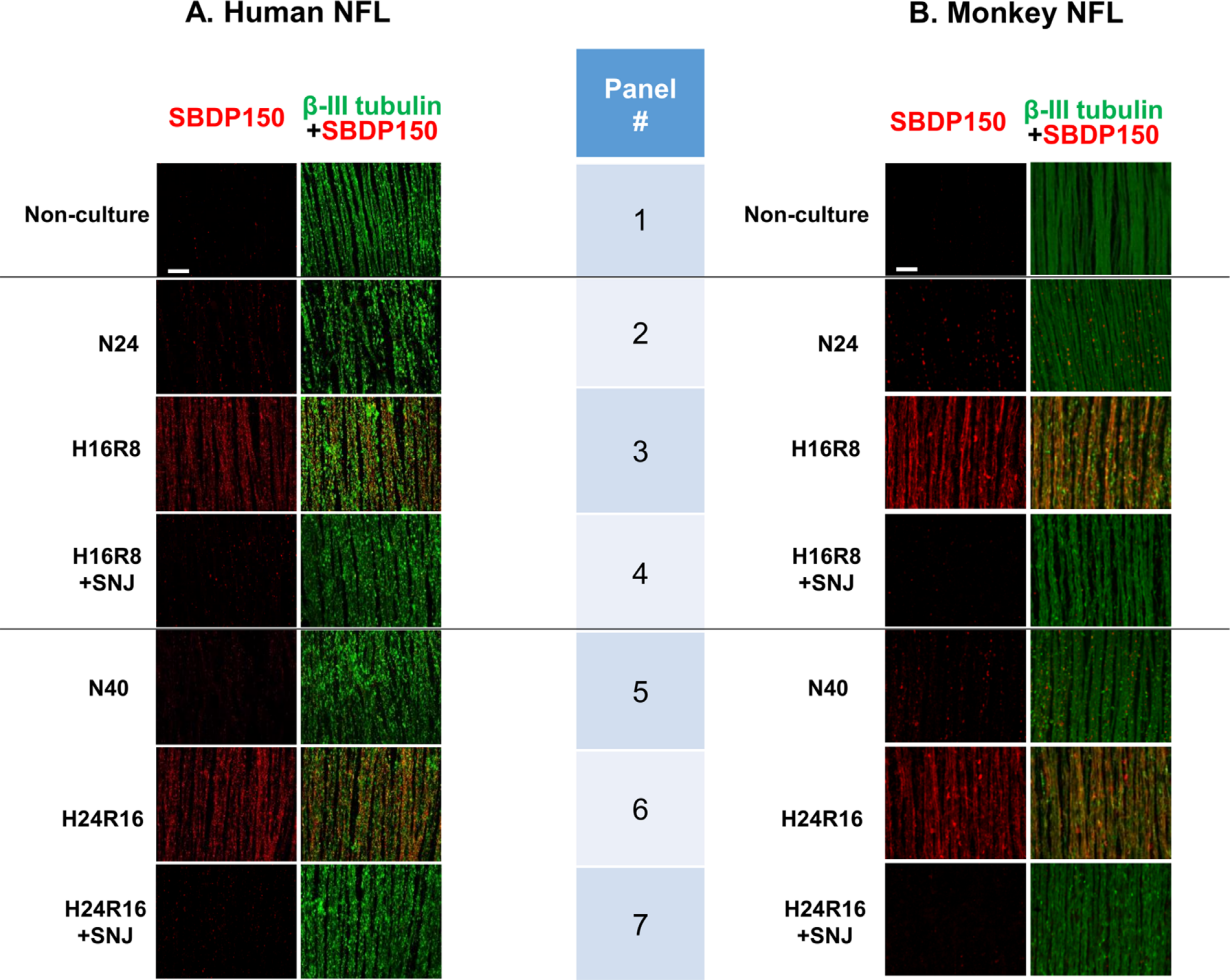
Calpain activation in β-III tubulin-positive, NFL from human (**A**) and monkey (**B**) retinal explants cultured under hypoxia/reoxygenation. Images show confocal microscopy of flat mounts stained with SBDP150 (*red*, *left columns*) that were then merged with those stained for β-III tubulin (*green*, *right columns*). LABELS: Non-culture, before culture; N24 or N40, normoxia for 24 or 40 hours; H16R8, 16 hours hypoxia/8 hours reoxygenation; H24R16, 24 hours hypoxia/16 hours reoxygenation; +SNJ, cultured with 100 µM SNJ-1945 under each condition. *Scale bar*: 50 µm.

Calpain activation was next measured in flat mount sections, where the distribution of calpain activity along the long fibers of NFL could be directly observed (Fig. 3). The marker protein for calpain activation SBDP150 (red) was densely apparent in human retina only after activation by hypoxia/reoxygenation (Fig. 3A, left column, Panels 3 and 6). The β-III tubulin (a neuron marker) colocalized with SBDP150, confirming activation of calpains in NFL (Fig. 3A, right column). SNJ-1945 attenuated calpain activation induced by hypoxia/reoxygenation in NFL (Panels 4 and 7). No obvious differences were observed between human and monkey retinas (Fig. 3B).

### Disruption of Membrane Barrier

**Figure 4.**
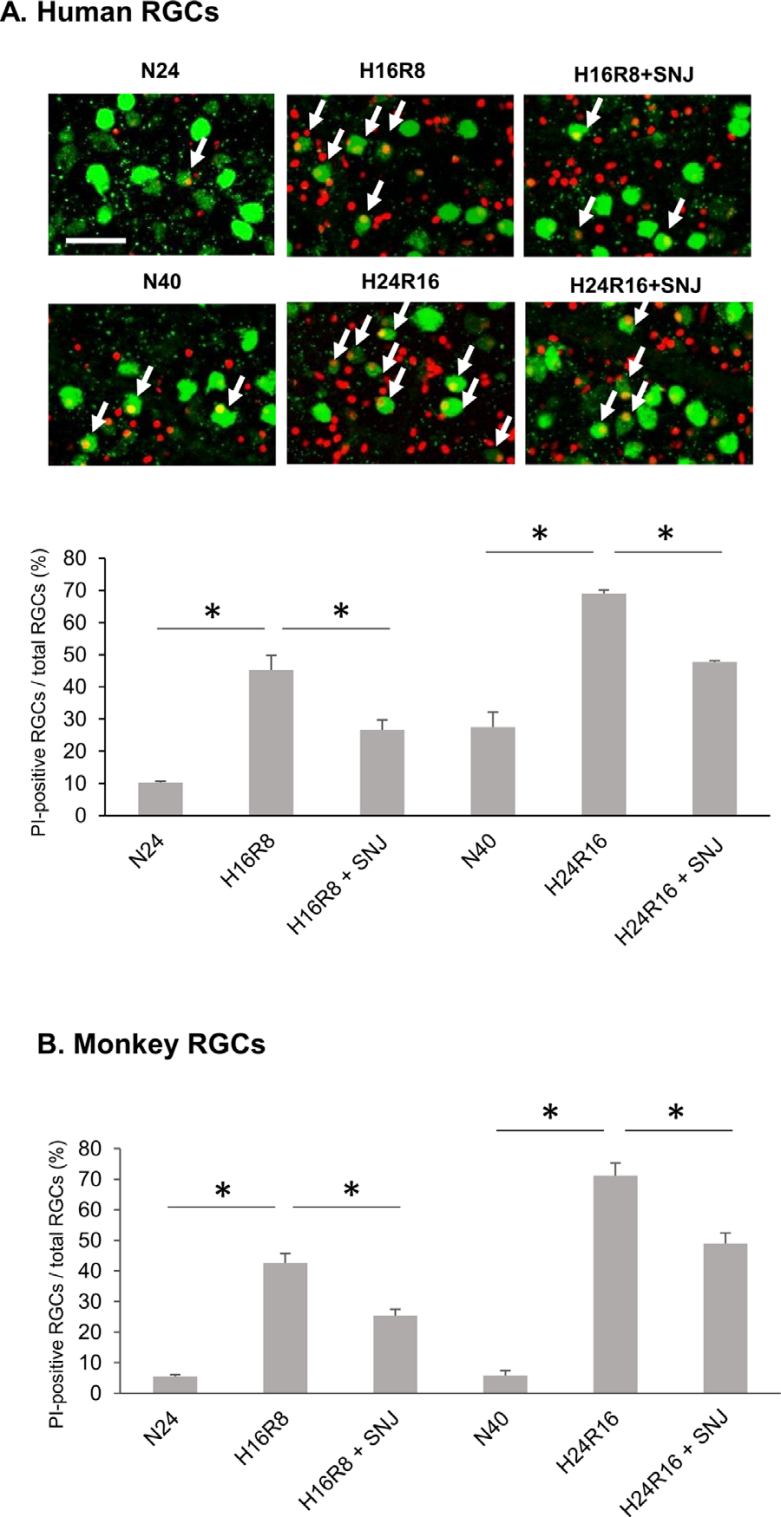
(**A**) Disruption of the membrane barrier and inhibition by calpain inhibitor SNJ-1945 in retinal ganglion cells (RGCs) from human retinal explants cultured under hypoxia/reoxygenation. (**Upper**) Images show GCL in flat mounts stained for influx of PI (*red*) and merged with the immunostaining for an RGC marker protein (RNA-binding protein with multiple splicing; RBPMS, *green*). PI-positive RGCs are indicated with *white arrows*. *Scale bar*: 50 µm. (**Lower**) Diagrams show higher percentage of PI-positive RGCs in the flat mount samples indicating membrane leakage. (**B**) Monkey retinal explants cultured under hypoxia/reoxygenation. Data are means ± SD (n = 3). *P < 0.05 (Wilcoxon rank sum test). LABELS: N24 or N40, normoxia for 24 hours or 40 hours; H16R8, 16 hours hypoxia/8 hours reoxygenation; H24R16, 24 hours hypoxia/16 hours reoxygenation; +SNJ, cultured with 100 µM SNJ-1945 under each condition.

The cytoskeletal protein α-spectrin was proteolyzed in human explants under hypoxia (Fig. 2A). Proteolysis of α-spectrin could result in loss of membrane integrity and allow influx of calcium. Loss of membrane integrity was directly tested by abnormal uptake of PI. The percentage of PI-positive cells increased in human retinal explants cultured under hypoxia/reoxygenation (Fig. 4A, arrows). SNJ-1945 ameliorated this increase (Fig. 4A), but effectiveness decreased with time. All results on membrane disruption by hypoxia in monkey retina (Fig. 4B) were similar to those in human, except that PI-positive cells did not increase in normoxic monkey retina with time (Fig. 4B, “N24” vs. “N40”).

### DNA Fragmentation

**Figure 5.**
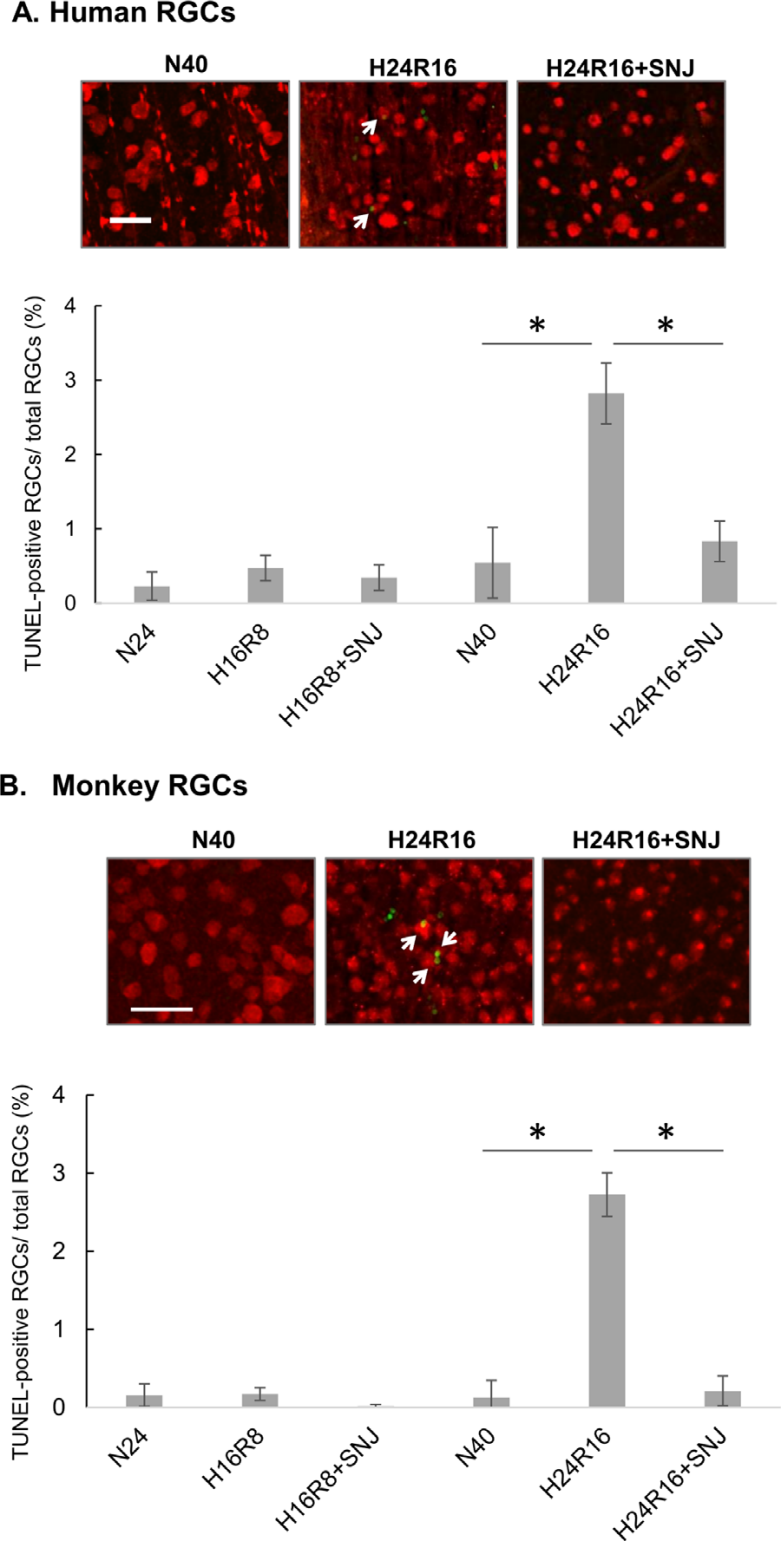
DNA fragmentation and inhibition by SNJ-1945 in retinal ganglion cells from human (**A**) and monkey (**B**) retinal explants cultured under hypoxia/reoxygenation. (**Upper**) Images show GCL in flat mounts stained with TUNEL (*green*) and merged with staining for RGC marker (RBPMS, *red*). TUNEL-positive RGCs are indicated with *white arrows*. *Scale bar*: 50 µm. (**Lower**) Diagrams show percentages of TUNEL-positive RGCs. Data are means ± SD (n = 3 for monkey, n = 5 for human). *P < 0.05 (Wilcoxon rank sum test). Labels are same as Figure 4.

Positive TUNEL staining indicates DNA fragmentation.^37^ We observed an increase in the percentage of TUNEL-positive RGCs after 24 hours’ hypoxia/16 hours’ reoxygenation in human and monkey retinas (Figs. 5A and 5B, arrows). The increase in TUNEL-positive cells was significantly inhibited by SNJ-1945. Note that the percentage of TUNEL-positive cells was only 3% of total RGCs. This was lower than in previous studies using gas-generating pouch system.^26^

### Hypoxia/Reoxygenation Does Not Activate the tAIF Caspase-Independent Pathway

DNA fragmentation is generally believed to be regulated by the executioner caspase-3.^38^ Yet caspase-3 seems not to be activated in our model of hypoxic retina (Fig. 2A, Blot no. 6). Thus a caspase-*in*dependent pathway was investigated. When other cell types are injured, AIF was truncated by mitochondrial calpain 1 to tAIF, translocated to the nucleus, where tAIF induced caspase-independent TUNEL-positive cells.^21^ Indeed, when lysates from our noncultured human and monkey retinas were incubated with exogenous calcium and purified human calpain 1, formation tAIF at 57 kDa was induced (Figs. 6A and 6B, lane 4). This was due to exogenous calpain 1 because addition of calpain inhibitor SNJ-1945 prevented tAIF formation (lane 5). Note, however, that solo addition of 10 mM Ca^2+^ was not sufficient to cause tAIF formation (lane 2, upper blots), even though α-spectrin breakdown was observed (lane 2, lower blots). Exogenous calpain 1 further degraded α-spectrin to 120 kDa (lane 4, lower blots), which was believed to be produced by activated caspase-3.^39^ Production of this breakdown product was prevented by SNJ-1945 (lane 5, lower blots) and caspase-3 inhibitor Z-DEVD (data not shown). Caspase-3 was activated by large amount of exogenous calpain 1 in the test tube, but caspase-3 was not activated in our explant model of hypoxic human retina.

**Figure 6.**
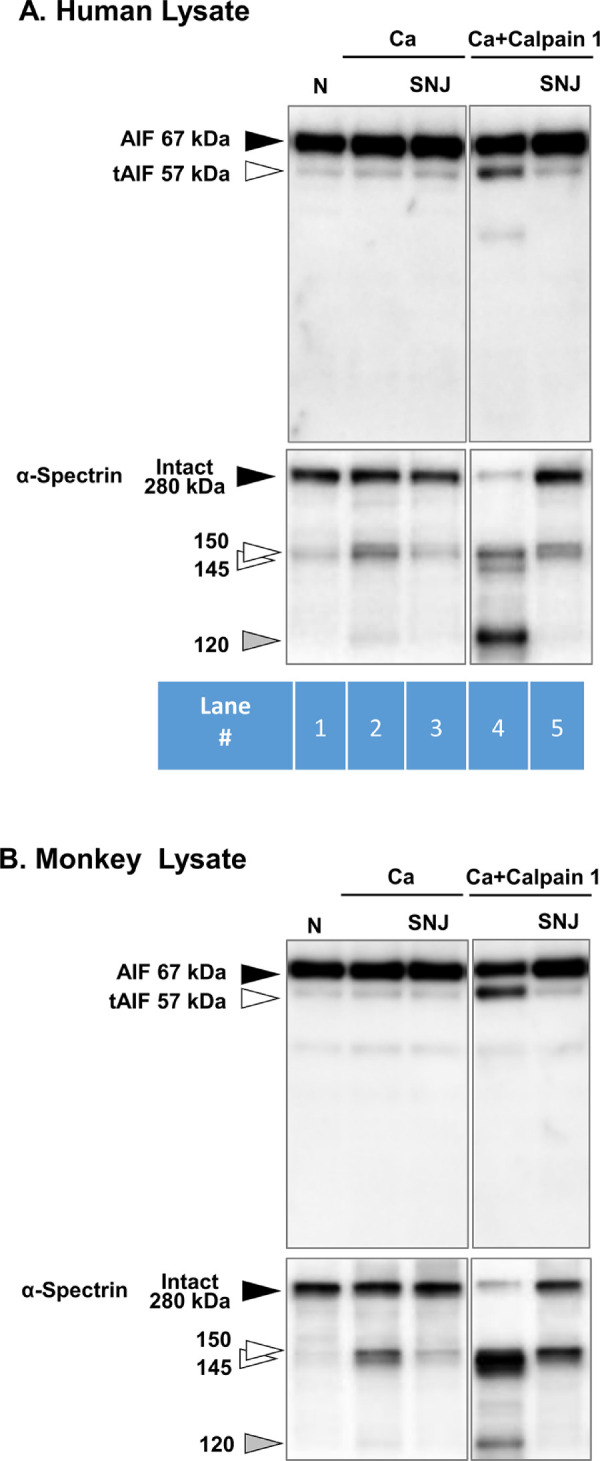
Production of truncated apoptosis inducing factor (tAIF) in human (**A**) and monkey (**B**) retinal lysates after incubation in the test tube for 30 minutes with exogenous calpain 1 and CaCl_2_ (lane 4). The tAIF band did not increase in human (**C**) or monkey (**D**) retinal explants cultured under hypoxia/reoxygenation (lanes 3 and 6). Calpain1, incubated with exogenous human calpain 1. Other labels are same as Figure 2.

**Figure 6.**
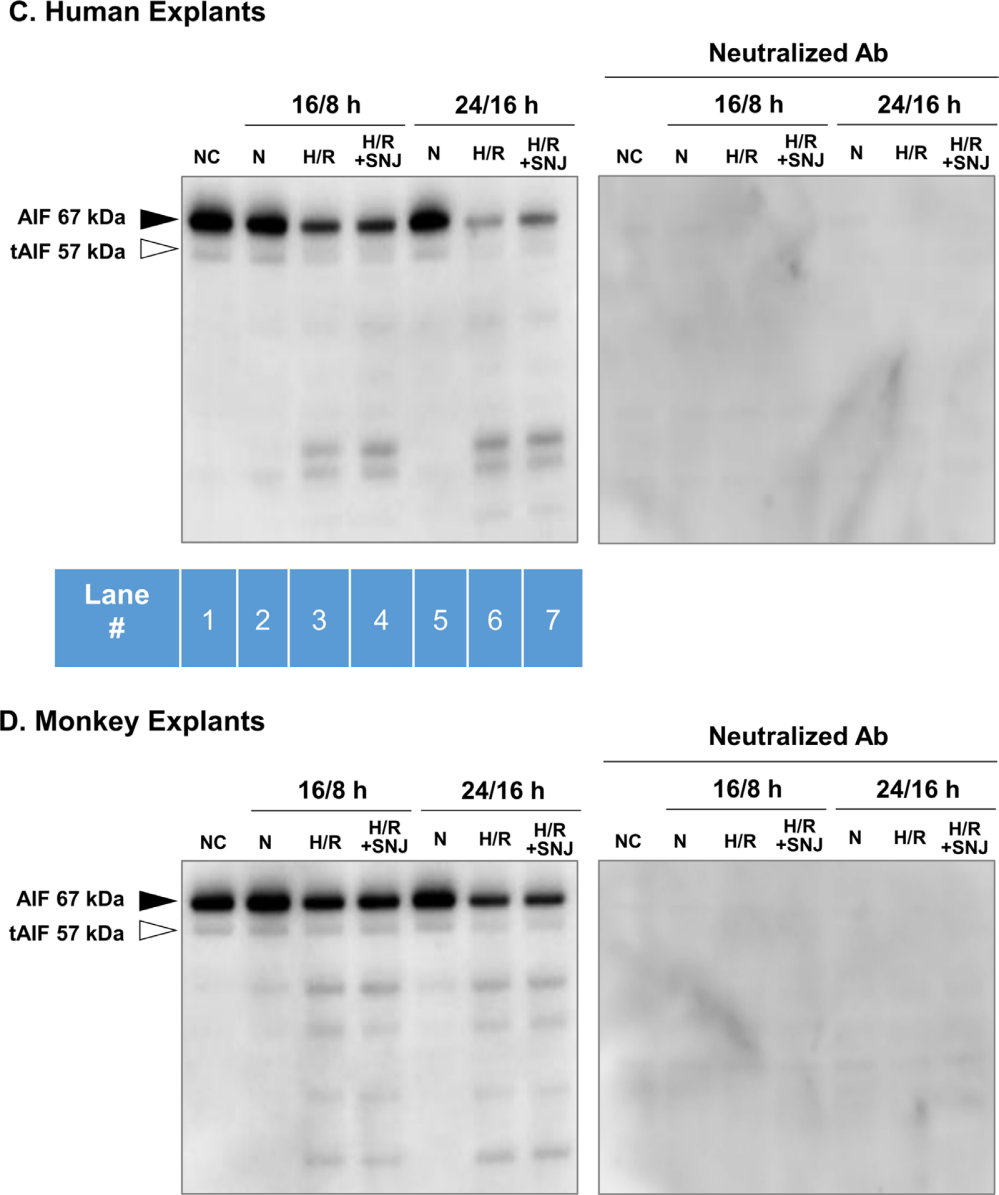
(Continued).

Under more physiological conditions where explants were exposed to hypoxia/reoxygenation, tAIF was not produced (Figs. 6C and 6D, left blots, lanes 3 and 6). As expected in the negative controls for this experiment, both AIF and tAIF immunostaining was abolished when the AIF antibody was first incubated with its immunizing peptide (Figs. 6C and 6D, right panels). Overall, our data indicated that although AIF was a theoretical substrate for large amounts of exogenous calpain 1 in the test tube; a tAIF-related, caspase-independent pathway was not the cause of DNA fragmentation in our explant model of hypoxic human retina.

## Discussion

### Calpain Activation in Human Retina

The most important finding of the experiments above was that the calpain system was activated in human retinal explants by hypoxia/reoxygenation. The calpain system was especially abundant in the NFL/GCL layers (Fig. 1). Note that calpains have been found in nearly all animals and tissues tested.^40^ Yet their specific functions pose an enigma. Calpains are essential for life—possibly by limited proteolysis of cell signaling proteins and during normal cell turnover.^41^ However, under conditions of abnormally high cellular calcium, excess calpain proteolysis leads to tissue damage and cell death.^41^^–^^43^ This later role seems to be the case in recent experiments where excess calpain activity has been observed in a wide variety of models of retinal ischemia utilizing rats,^16^^,^^23^^,^^44^ mice,^45^ and monkeys.^25^^,^^26^ The present findings of calpain activation in human retinas (Fig. 2A) are important because an orally available calpain inhibitor is currently in human trials (Trial ID: jRCT2021190013). This will be especially relevant if excess, hypoxia-induced calpain activity is found to be significant in diseases such as retinal artery occlusion, glaucoma, and diabetic retinopathy.

Our previous study showed that calpain-specific SBDP150 fragment is predominantly observed in hypoxic NFL/GCL layers, where both calpains 1 and 2 were localized.^26^ In the present experiment, SBDP150 appeared in hypoxic NFL followed by disruption of membrane barrier and DNA fragmentation in GCL (Figs. 3^4^–5). Because SBDP150 is produced in retinal lysates incubated with calpains 1 and 2 (data not shown), both calpain 1 and 2 may contribute to proteolysis of α-spectrin and RGC degeneration in hypoxic NFL/GCL, although the relative contribution of each calpain is not clear. This may be elucidated by in situ measurement of specific active calpain forms in future studies.

A relatively high calpastatin to calpain ratio was observed in human retina,^28^ whereas calpastatin is also a known substrate for calpains.^34^ Proteolysis of calpastatin would allow activation of calpains. In hypoxia/reoxygenation-induced retinal explants, degradation of calpastatin and activation of calpain were observed. SNJ-1945 only partially inhibited degradation of calpastatin. SNJ-1945 also inhibited autolysis of calpain 1, but did not inhibit autolysis of calpain 2 (Fig. 2A). This difference may be due to localization of calpain 2 in the INL. Degradation of calpastatin could have occurred by calpains released from an inactive calpastatin/calpain complex, and then inhibited by SNJ-1945 in the NFL.

Activated calpain proteolyzed α-spectrin in the NFL (Fig. 3), and more PI-positive cells were observed (Fig. 4). Degradation of scaffold protein α-spectrin probably caused disruption of membrane integrity (Fig. 4). Membrane disruption was inhibited by SNJ-1945, but it was only partially effective and diminished with time. This suggested that other factors cooperated in membrane disruption.

Caspase-3 causes DNA fragmentation in several types of cells,^38^^,^^46^^,^^47^ but caspase-3 was not activated in our hypoxic retinal explants (Fig. 2). The tAIF is known to induce DNA fragmentation in a caspase-*in*dependent pathway. AIF is localized in the mitochondrial membrane and intermembrane spaces, and calpain 1 converts AIF to tAIF.^21^^,^^22^ However, tAIF was not observed in our hypoxia/reoxygenation-induced explants. This result suggested that calpain may not be activated in the mitochondria; only cytosolic calpain was activated and proteolyzed α-spectrin. We do not know whether calpain regulation by calpastatin is different in the cytosol compared to mitochondria. However, a recent study with mitochondrial lysate containing 14.3 nM calcium found that calpastatin associated with calpain 1.^48^ This may indicate that mitochondria contain an inactive calpain 1/calpastatin complex.

### Significance

The present experiments attempted to determine the significance of hypoxia-induced calpain activation on pathologic changes in observed in human retina. Calpains 1 and 2 were activated by hypoxia/reoxygenation even in the presence of the endogenous inhibitor calpastatin (Fig. 3A). Calpain activation was associated with increased α-spectrin breakdown (Fig. 2A), membrane permeability (Fig. 4A), and DNA fragmentation (Fig. 5A). Although DNA fragmentation has been associated with caspase-3, neither caspase-3 activation (Fig. 2A) nor truncated AIF production (Fig. 6C) was increased by hypoxia/reoxygenation. Note that the synthetic inhibitor SNJ-1945 was able to penetrate the organ cultured retinal explants and inhibit calpain-induced proteolysis (Figs. 2^3^,^4^,^5^–6). All these results were verified in a second primate model - monkey explants (Figs. 2C, 3B, 4B, 5B, and 6D).

**Figure 7.**
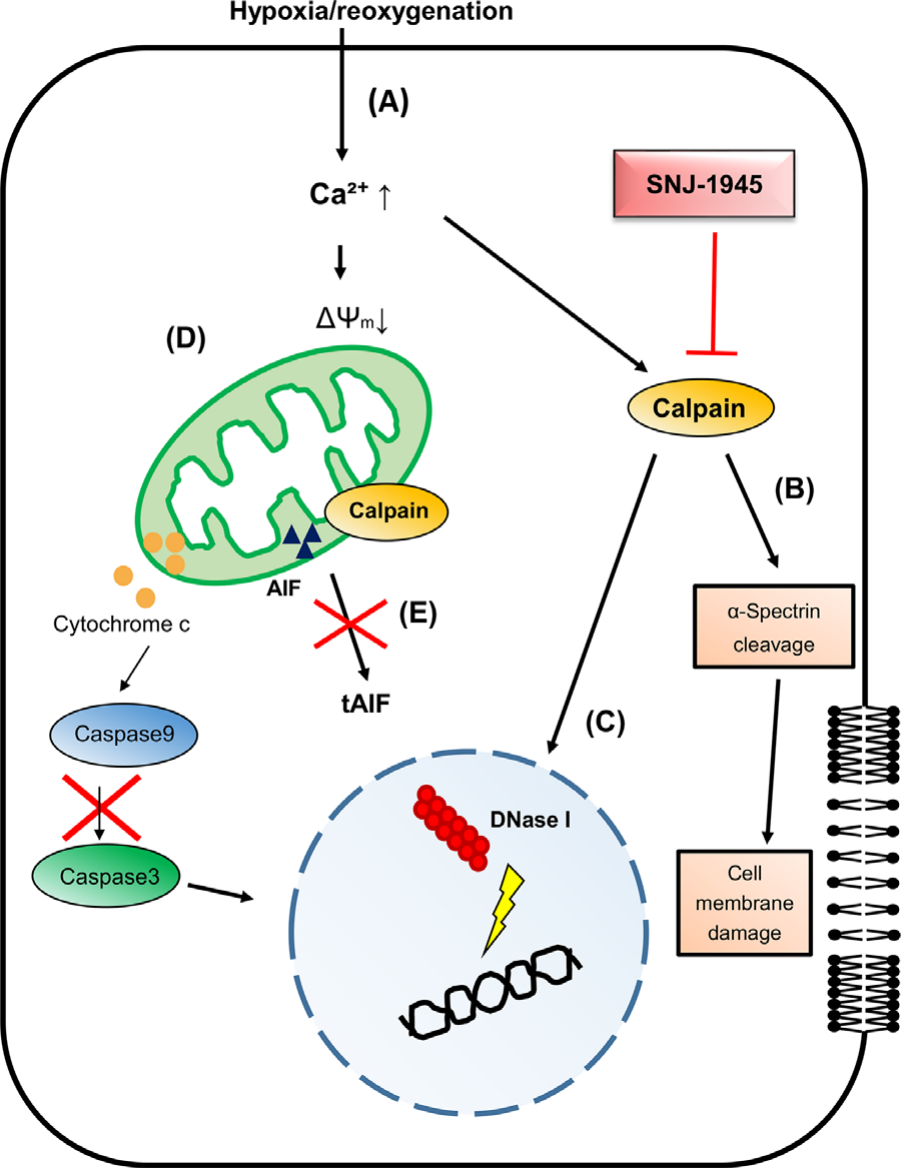
Hypothesis for the role of activated calpain in hypoxic retina. (**A**) Plasma membrane damage by hypoxia allows excess calcium influx. (**B**) Calcium activates cytosolic calpain and proteolyzes α-spectrin, leading to further disruption of plasma membrane. (**C**) In the nucleus, activated calpain also proteolyzes hamper proteins (e.g., actin) and releases DNase I, followed by DNA fragmentation. (**D**) In mitochondria, elevated calcium changes the membrane potential and releases cytochrome c, potentially activating caspase-9 and -3 sequentially. Note that caspase-3 is not activated in our current model of hypoxic retina. (**E**) Fragmentation of DNA and cell death may be triggered by calpain activation, but not direct action via the AIF pathway.

We speculate that these changes are related as shown in Figure 7: (A) Plasma membranes are damaged by hypoxia and this allows influx of excess calcium. Hypoxia in humans is certainly a possibility from such factors as aging, increased intraocular pressure, retinal vessel occlusions, elevated blood glucose, environmental toxins, and deleterious genetic conditions. (B) Calcium activates cytosolic calpain and proteolyzes α-spectrin, leading to further disruption of plasma membrane. (C) In the nucleus, activated calpain also proteolyzes hamper proteins (e.g., actin) and releases DNase I, followed by DNA fragmentation.^49^ (D) In mitochondria, elevated calcium changes the membrane potential and releases cytochrome c to activate caspase-9. Note that caspase-3 is not activated in this model. (E) Fragmentation of DNA and cell death may be triggered by calpain activation, but not by direct action on AIF.

### Limitations

Limitations include using a model where the explants have no functioning blood supply to deliver nutrients, to remove waste products, and to help maintain normal penetration of substances into the multilayered retina. It is hoped that the abundant culture media and noted lack of pH changes would mitigate such problems. Better control of the atmosphere in the hypoxic chamber did seem to produce a more useful and relevant culture model for the human situation.

The viability of the explants was limited to two days compared to decades of exposure of intact eyes to various insults. The short duration is an unfortunate reality of current organ culture with the delicate retina and its high metabolic activity. Lack of direct measures of calcium concentrations in specific retinal layers of retina in our experiments is a drawback that may be solved in future experiments using Ca^2+^-indicating reagents. Although absolute concentrations are unknown, we safely assumed that calcium levels were quite elevated because both µM-requiring calpain 1 and mM-requiring calpain 2 were obviously activated.

### Prospect

Rodents are commonly chosen for many studies evaluating drug efficacy, although preclinical data performed in rodent models sometimes fails to translate to the human situation. The poor translation from rodents to humans is not surprising, because of anatomic and functional differences in species. Here we demonstrated that our human organ culture model could be useful for evaluating drugs in retinal hypoxic diseases. We hope our model contributes to successful translation of predictive preclinical assessment of new drugs to clinical use and accommodates future unmet medical needs.
